# 

**Published:** 1889-02-09

**Authors:** 


### CONTENTS.

**Give It a Fair Trial** - 291

**Editor's Letter Box.**—A Suggestion for Secretaries, etc. - 292

**The Doctor's Pulpit.**—"All Sorts and Conditions" of Women - 293

**Short Sight and Its Causes** - 294

**The Highroads to Health** - 295

**The Holloway Sanitorium for the Insane at Virginia Water, Egham** - 296

**Babies.**—VI. Queer Babies - 297

**Notes and News** - 298

**Everybody's Page** - 300

**Annotations.**—"Extract of Horse"—"After Dinner Walk a Mile"—"Dried-up" Pensioners - 301

**Physiology and Its Uses.**—Fat - 302

**Voyages in Vacation.**—XVIII. - 303

**False Impressions.** By CAROLINE FOTHERGILL, Author of "An Enthusiast," "A Voice in the Wilderness," etc. Chapter XVIII - 304

**Scraps and Gleanings** - 306

**Amusements and Relaxation** - 306

**EXTRA SUPPLEMENT—THE NURSING MIRROR.**

**En Passant.**—News from the Provinces—Women's Wages—Unintelligent Nursing.—The First Thousand — Workhouse Nursing—The Duke of Westminster - lxxiii
**Original Articles.**—Case Number Nine - lxxiv
**Common Abbreviations** - lxxv
**Nurses as Stewardesses.**—The Pros and Cons - lxxv
**Everybody's Opinion.**—A Correction—Rest and Exercise—Nurses as Stewardesses—A Lack of Courtesy—The Pension Fund - lxxvi
**Appointments, Lectures, Vacancies** - lxxvi

